# Extracellular vesicles as bioactive nanotherapeutics: An emerging paradigm for regenerative medicine

**DOI:** 10.7150/thno.72812

**Published:** 2022-06-21

**Authors:** Min Li, Fang Fang, Meng Sun, Yinfeng Zhang, Min Hu, Jinfeng Zhang

**Affiliations:** 1 Key Laboratory of Molecular Medicine and Biotherapy, School of Life Sciences, Beijing Institute of Technology, Beijing 100081, P. R. China.; 2 International Medical Center, Beijing Friendship Hospital, Capital Medical University, Beijing, 100050, P. R. China.; 3 Department of Hepatobiliary Surgery, Jinan University First Affiliated Hospital, Guangzhou, 510630, P. R. China.

**Keywords:** extracellular vesicles, exosomes, nanotherapeutics, tissue regeneration, regenerative mechanisms, EV engineering strategy

## Abstract

In recent decades, extracellular vesicles (EVs), as bioactive cell-secreted nanoparticles which are involved in various physiological and pathological processes including cell proliferation, immune regulation, angiogenesis and tissue repair, have emerged as one of the most attractive nanotherapeutics for regenerative medicine. Herein we provide a systematic review of the latest progress of EVs for regenerative applications. Firstly, we will briefly introduce the biogenesis, function and isolation technology of EVs. Then, the underlying therapeutic mechanisms of the native unmodified EVs and engineering strategies of the modified EVs as regenerative entities will be discussed. Subsequently, the main focus will be placed on the tissue repair and regeneration applications of EVs on various organs including brain, heart, bone and cartilage, liver and kidney, as well as skin. More importantly, current clinical trials of EVs for regenerative medicine will also be briefly highlighted. Finally, the future challenges and insightful perspectives of the currently developed EV-based nanotherapeutics in biomedicine will be discussed. In short, the bioactive EV-based nanotherapeutics have opened new horizons for biologists, chemists, nanoscientists, pharmacists, as well as clinicians, making possible powerful tools and therapies for regenerative medicine.

## Introduction

Regenerative medicine aims at the structural restoration and functional reestablishment of impaired or missing tissues by replenishing, replacing or repairing cells, tissues and organs, holding great promise for human healthcare 1. Particularly, regenerative medicine can substantially cure diseases including heart disease, nerve injuries and diabetic wounds once poorly managed with conventional drugs or current treatment procedures. In recent decades, there are three main therapies in regenerative medicine, such as stem cell therapy, bio-degradable scaffold utilization, and material-based approach 2,3. Among them, stem cell therapy has been considered as the most promising modality for tissue regeneration because its potential for rapid cell growth and differentiation in damaged organs by using the stem cells with high self-renewal and rapid proliferative capabilities 4,5.

Although effective, there are considerable limitations restricting the wider clinical application of stem cell therapy, including 1) biosafety concerns deriving from abnormal differentiation, immune rejection and tumorigenic risk 6-9, 2) systemic administration challenges such as long-term viability and differentiation capability, *in vivo* stem cell proliferation and targeted delivery to the injured sites 10, 3) handling issues on cell storage and transport 11. On the other hand, mounting evidence has indicated that the biological function of stem cells is in large part attributed to their secreted extracellular vehicles (EVs) which can modulate the damaged tissue microenvironment through a paracrine mechanism, thus ultimately inducing cell differentiation and organogenesis 12,13. In light of the above considerations, EVs have emerged as a superb alternative strategy to the traditional stem cell therapy for the regenerative medicine applications.

**Figure 1:**
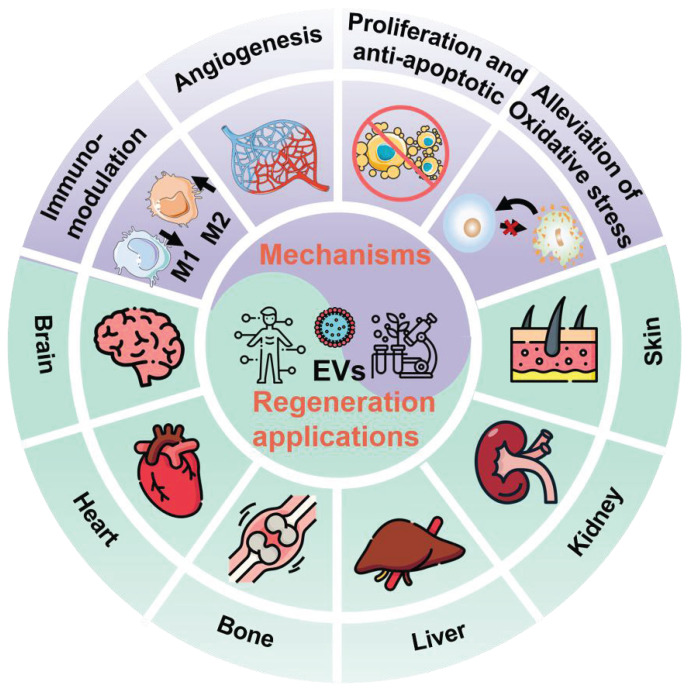
Schematic illustration of the EVs for regeneration applications in various organs including brain, heart, bone, liver, kidney, and skin as well as the corresponding therapeutic mechanisms.

In general, EVs, typically including exosomes, microvesicles (MVs), and apoptotic bodies based on their size and biogenesis, have originally been considered as a disposition apparatus for waste materials from cells, but are now recognized as biological cell-secreted nanoparticles (30-2000 nm) enclosed by double phospholipid membranes, which play important roles in intercellular communication 14. Because EVs contain a vast array of biomolecules, such as lipids, proteins, nucleic acids (*e.g.*, microRNA (miRNA), messenger RNA (mRNA), and DNA), as well as soluble small molecules inherited from their parental cells 15, which are involved in various physiological and pathological processes including cell proliferation, immune regulation, angiogenesis and tissue repair, making EVs being considered as one of the most attractive nanotherapeutics for regenerative medicine (**Figure 1**). Indeed, EVs exhibit great superiority over the conventional stem cell therapy for regenerative medicine, such as, multiple biologically therapeutic effects, exogenous cargo delivery functions, enhanced biocompatibility and biosafety, as well as improved reproducibility and stability 16.

Despite EVs having shown considerable potential in regenerative medicine application, their development is still at an early stage, where more attention should be devoted to both fundamental research and clinical practices. Herein, we provide a systematic review of the latest progress of EVs for regenerative medicine. The biogenesis, function and isolation technology of EVs will be introduced firstly. Then, the underlying therapeutic mechanisms of the native unmodified EVs as well as engineering strategies of the modified EVs as regenerative entities will be discussed. Afterwards, a specific focus will be placed on the tissue repair and regeneration applications of EVs on various organs including brain, heart, bone and cartilage, liver and kidney, as well as skin. More importantly, current clinical trials of EVs for regenerative medicine will be briefly presented. Finally, the remaining challenges and some insightful perspectives of the currently developed EV-based nanotherapeutics in biomedicine will be briefly highlighted, which will be beneficial to the future development of the EVs or other bioinspired nanotherapeutics. We believe that this review will appeal to different researchers of biologists, chemists, nanoscientists, pharmacists, clinicians as well as scientists from interdisciplinary fields.

## Biogenesis, Functions, and Isolation Technology of EVs

### Classification and biogenesis of EVs

More than half a century has passed since EVs were discovered as “platelet-dust” in 1967 17. Afterwards, the terms “extracellular vesicle” and “exosome” were respectively defined in 1971 and 1981 18,19. In 1983, transferrin carrier as the first biological function of EVs was reported 20. For several decades, the EVs have been greatly developed from native biologicals 21 to engineered small molecule drug delivery systems 22, from cancer vaccines 23 to regenerative entities, from preclinical research 24 to clinical trials 25. During this period of EV development, the classification and biogenesis mechanisms of EV subtypes have been gradually clear.

**Figure 2:**
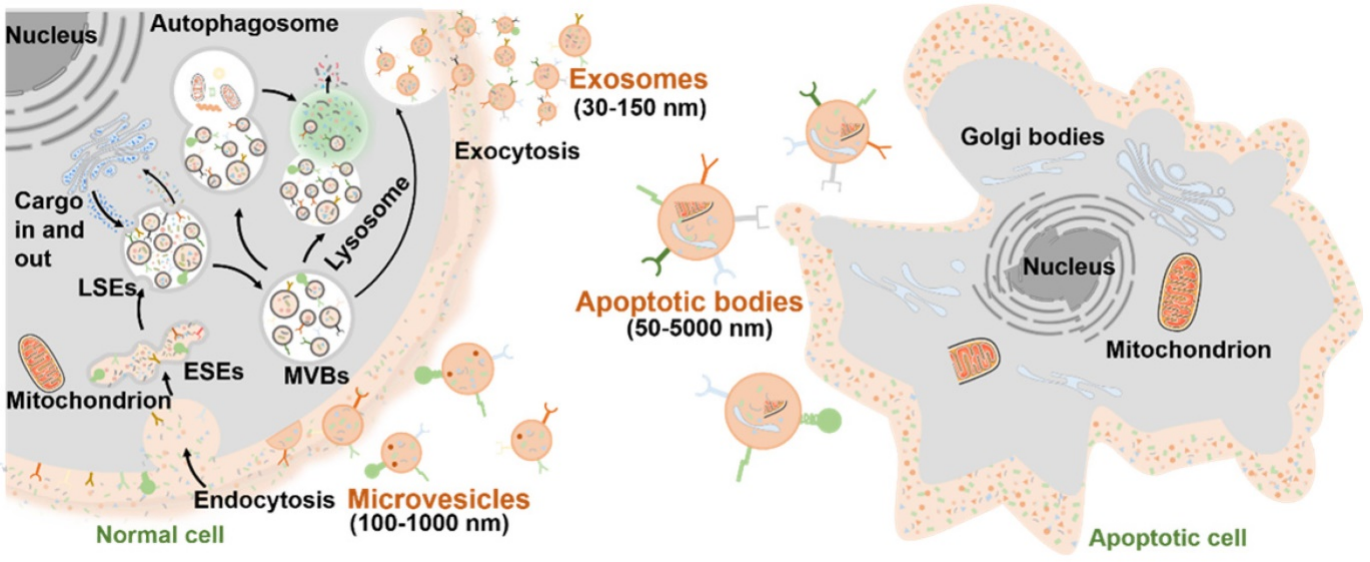
Biogenesis of three major subtypes of EVs including exosomes, microvesicles and apoptotic bodies.

As mentioned above, the EVs are normally divided into three major subtypes (**Figure 2**): 1) Exosomes are small vesicles (30-150 nm) secreted from the plasma membrane which go through invagination and inward budding, ultimately exhibiting a typical cup-shaped structure; 2) Microvesicles (100-1,000 nm) are formed by directly budding from the cell membrane; 3) Apoptotic bodies (50-5,000 nm) are budded from the plasma membrane of apoptotic cells with shrinkage and fragmentation. The biogenesis mechanisms of EVs involve a series of endocytosis and exocytosis. During the formation of exosomes, twice invagination of the plasma membrane occurred. For the first time, membrane proteins and extracellular soluble molecules are enclosed in the cup-shaped structure, which further forms the early-sorting endosomes (ESEs) and late-sorting endosomes (LSEs) 26. After the invagination of the LSE membrane, numerous multivesicular bodies (MVBs) containing multiple intraluminal vesicles (ILVs) are generated, which is also called the second invagination of plasma membrane. MVBs can fuse with the plasma membrane to eventually release the ILVs outside the cell as exosomes with a diameter of 30 to 150 nm. Meanwhile, the release of the microvesicles may be attributed to a calcium-influx-activated asymmetrical redistribution of the phospholipids in the cell lipid bilayer, which promotes inward budding of the cell membrane 27. However, the molecular mechanisms involved in microvesicles formation are still needed to be further explored. On the other hand, the apoptotic bodies are more like the fragments of dying cells, which are formed by blebbing of plasma membrane from cells that undergo apoptosis. Similar to the microvesicles, the specific biogenetic process of apoptotic bodies is still unclear, which is worth making deeper studies in the future. It is worth noting that the biogenesis of EVs is a dynamic process, and the internal components of EVs released in the same spatiotemporal situation may also be inconsistent both *in vivo* and *in vitro*. Moreover, the EV biogenesis may also be regulated by different cell types, culture conditions, and cell sources 28.

### Components and applications of EVs

EVs contain a variety of bioactive substances such as proteins, lipids, nucleotides and some soluble metabolites. Extensive research has revealed that the proteins of EVs usually include membrane proteins, cytoplasmic and nuclear proteins. Some specific proteins present on EVs such as Alix, TSG101 and tetraspanins (transmembrane proteins) CD9, CD63 and CD81 can serve as characteristic biomarkers for the identification of EVs. It is noteworthy that some organelles and their associated proteins may also be enriched in EVs under certain specific conditions. For example, some EVs selectively package mitochondria as well as mitochondria-related proteins or nucleic acids and subsequently become mitochondria-rich EVs 29,30. Furthermore, the nucleotides of EVs generally include DNA, mRNA, retrotransposons, small interfering RNAs, and other non-coding RNAs 31, while the lipids within EVs usually include glycerophospholipids, sphingolipids, and cholesterol 32. It was also discovered that the variation of the EV composition largely depends on their origin, their specific intracellular release sites as well as their physiological or pathological state. For example, 80-90% of the lipids are phosphatidylcholine in EVs derived from B16-F10 melanoma cells, while only 60% is phosphatidylcholine and 30% is sphingomyelin in EVs derived from human metastatic pancreatic adenocarcinoma cells 32. Even the same tissue-secreted EVs may contain entirely different components, which are known as EV heterogeneity 33. This issue is a critical task for moving EVs' research forward. Therefore, the exact proportions of different components in specific EVs deserve to be further investigated in depth. As of May 2022, there are 349,988 proteins, 38166 RNA, and 639 lipids in the statistics of the Vesiclepedia database (http://www.microvesicles.org/), which is one of the most predominant EV databases generally available.

**Figure 3:**
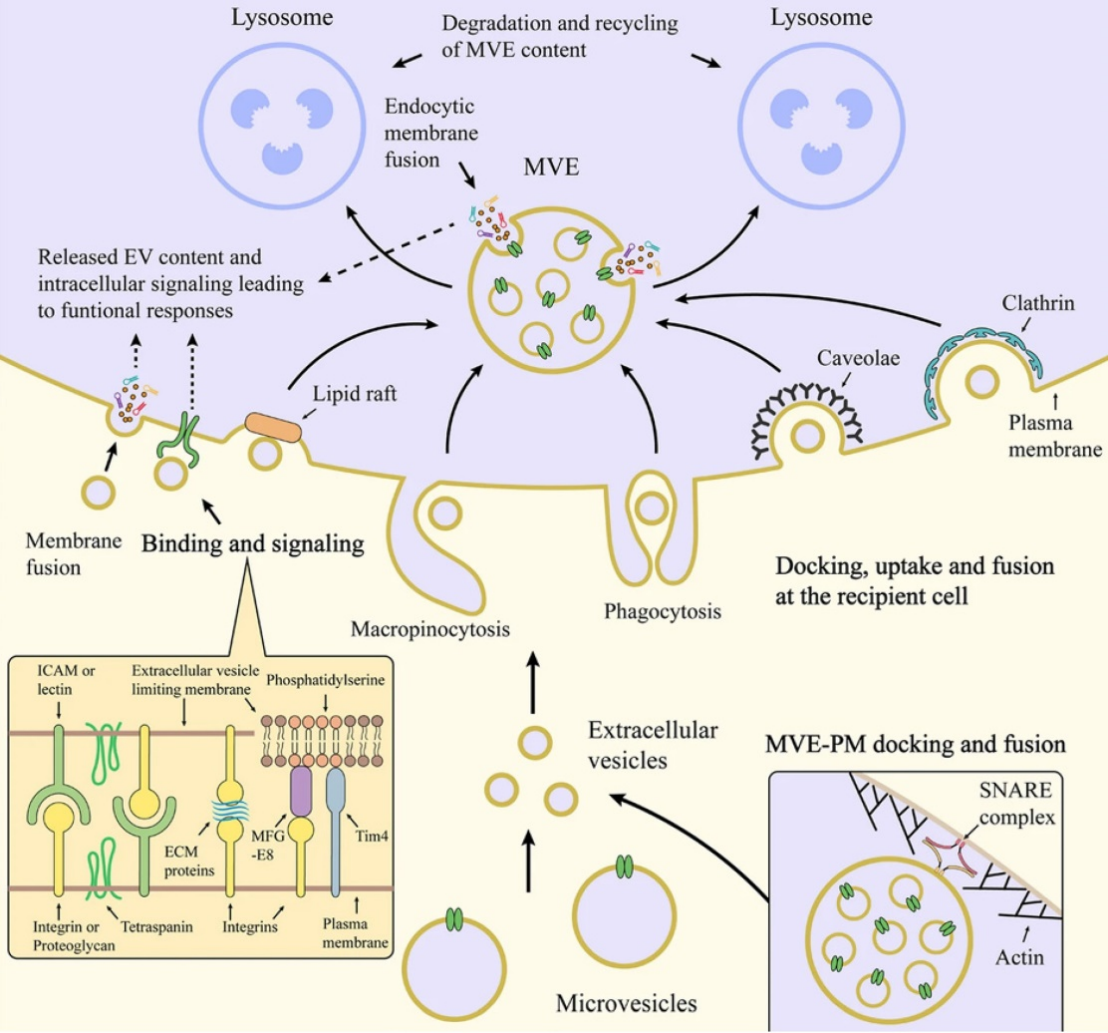
Schematic illustration of interaction between EVs and target cell membrane 40. Copyright 2020, Springer Nature.

EVs typically interact with the membrane of target cells in three ways once released extracellularly: receptor-ligand interaction, endocytosis, or membrane fusion to internalize EVs (**Figure 3**). Through binding of the specific ligand from EVs (such as tetraspanins, ICAM, lectins, and phosphatidylserine) and their corresponding affinity receptors on the target cell surface (such as integrins, heparan sulfate proteoglycans, and lipid-binding proteins), EVs can dock on the target cell surface and further trigger intracellular signaling pathways 34. One of the consequences of the triggered signaling pathways is to enable clathrin-mediated endocytosis of EVs *via* specific receptor-ligand interaction 35. Meanwhile, EVs can also be internalized into target cells through other endocytic pathways, such as phagocytosis, macropinocytosis, caveolae- or lipid rafts-mediated 36,37. For example, exosomes derived from PC12 cells (rat adrenal medulla tumor differentiated cell line) are more dependent on clathrin-dependent uptake by endocytosis 38. In addition, EVs that are docked at the plasma membrane can release their contents into the cytoplasm of recipient cells by membrane fusion 34. In fact, the mechanisms of interaction are mainly decided by the source, properties and membrane composition of the EVs, and the nature of the target cells 39. Once EVs successfully interacted with the target cells in one or more specific ways, EVs-containing bioactive cargos would be transferred from the EVs to the target cells and trigger a series of subsequent signaling pathways. In this context, EVs are indeed key modulators of physiological responses in intercellular communication under either normal or pathological conditions.

Currently, the applications of EVs can be predominantly divided into two main aspects: diagnosis and treatment of diseases. On one hand, EVs existing in all kinds of body fluids of animals and microorganisms can reflect the state of their parent cells. In a healthy state, EVs and their included components maintain a constant basal level to keep homeostasis. Noteworthy, the disordered secretion and abnormal content of EVs are related to the deviation of physiological indexes from the body's normal range. Therefore, EVs can be used as an important biomarker for the diagnosis and prognosis of diseases. At present, EVs have been widely used in the diagnosis of pancreatic malignancy, prostate cancer, neuropathies, pregnancy disorders, *etc.* 41,42. Besides, native EVs acting as intercellular communication factors can regulate immune responses, cell proliferation and differentiation, angiogenesis, anti-apoptosis, as well as oxidative stress, leading to playing an important role in tissue regeneration, which will be explained in detail below.

On the other hand, the naturally-formed and nano-sized EVs show several inherent advantages over conventional synthesized nanoparticles, such as excellent biocompatibility, less immunogenicity, increased stability in the blood, high penetration depth in deep tissue, and the ability to target specific disease sites *via* their homing characteristics 43. As such, EV-based nanosystem has attracted tremendous attention directed toward their applications for drug delivery. For example, EVs from blood with the natural brain-homing ability through the transferrin-receptor-mediated interaction can be applied to deliver Parkinson-related drugs across the blood-brain barrier 44. In addition, the membrane of EVs can be further chemically or genetically engineered to improve their targeting capability 45. Up to now, EVs have made remarkable advances in the treatment of regenerative medicine-related diseases and cancer 46.

### Isolation technology of EVs

**Figure 4:**
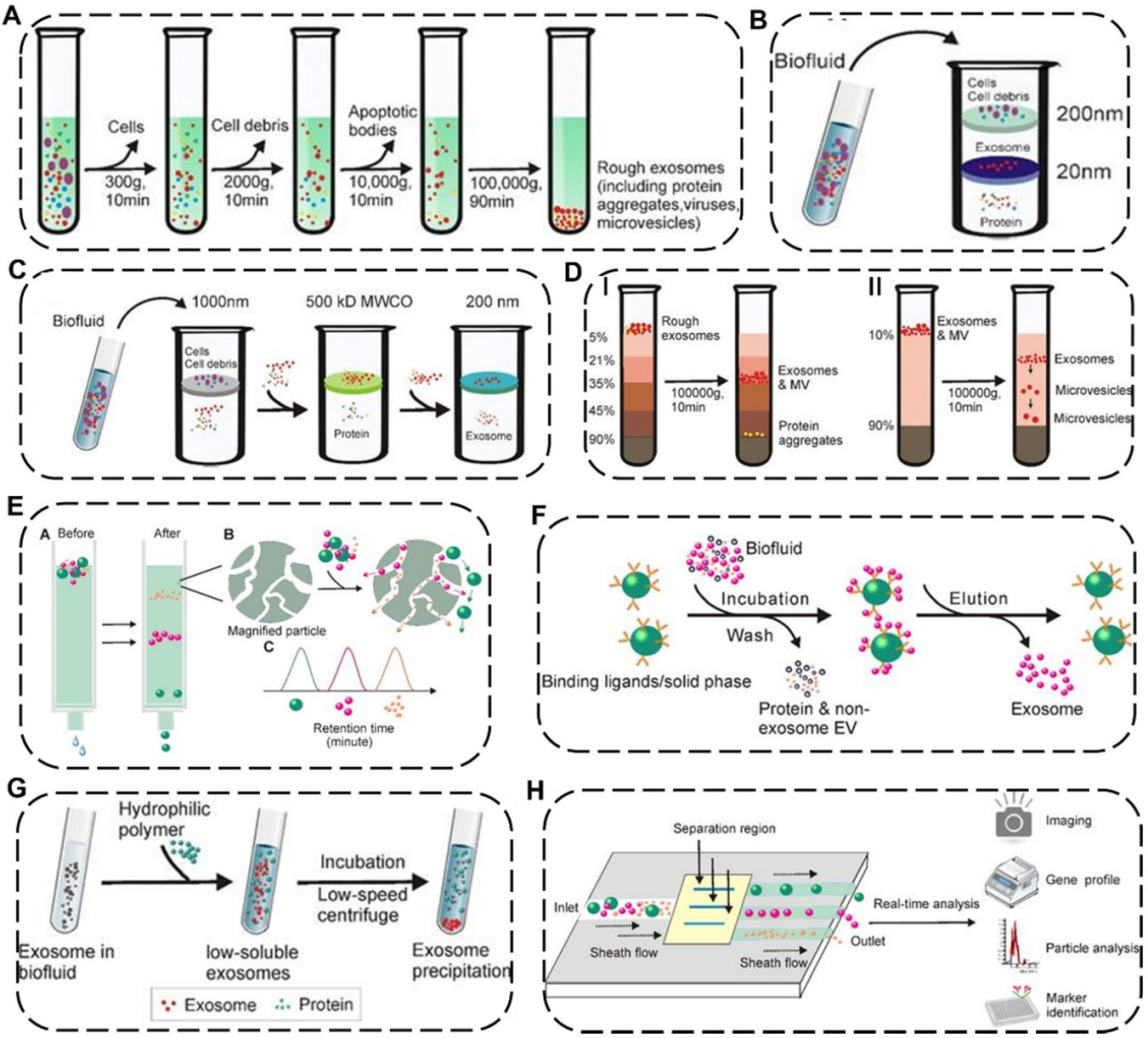
** Schematic illustration of representative isolation technology of EVs. (A)** Differential ultracentrifugation. Density-gradient centrifugation including **(B)** isopycnic density-gradient ultracentrifugation and **(C)** moving-zone gradient ultracentrifugation. **(D)** Ultrafiltration including I) tandem ultrafiltration and II) sequential ultrafiltration. **(E)** Immunoaffinity capture. **(F)** Size-exclusion chromatography. **(G)** Polymer Precipitation. **(H)** Microfluidic techniques. Reproduced with permission 48. Copyright 2020, Ivyspring International Publisher.

EVs are nano-to-micro sized extracellular vesicles distributed in various complex fluid environments *in vivo*, thus indicating that the standardized isolation technology of EVs is exceptionally challenging 47. As a single method unsuitable for all types of EV samples, more and more efforts have been made to explore newly efficient but universal approaches according to the physicochemical properties of EVs. Up to now, many separation strategies of EVs have been reported, including differential ultracentrifugation, density-gradient centrifugation, ultrafiltration, immunoaffinity capture, size-exclusion chromatography, polymer precipitation, fluidic techniques with distinctive preponderance and weaknesses for each approach (**Figure 4 and Table 1**) 48.

Among these separation and purification techniques, differential ultracentrifugation and density-gradient centrifugation are the two most widely used traditional approaches and are considered the “gold standard” for EV isolation. Differential ultracentrifugation stepwise removes different components (*e.g.*, cell debris and organelles) from cell culture media or biological fluids containing exosomes by numerous cycles of centrifugation with speed from 300 ×g to 100,000 ×g at 4 °C 49. However, the shortcomings of differential ultracentrifugation include time-consuming, low recovery rate, and sediment with other biological impurities. To remove the impurities from EVs, density-gradient centrifugation could be preferably selected where the extracellular components could stay in the position of the medium with similar density by gravitational or centrifugal force fields and subsequently be separated from EVs 50. Although this method has the potential for obtaining EVs with high purity, it also faces a time-consuming issue. By contrast, ultrafiltration can save time by using different molecular weight cut-offs ultrafiltration membranes for selective separation of samples, which could be divided into tandem ultrafiltration and sequential ultrafiltration. However, shear stress applied in the ultrafiltration process may lead to changes in the potential physical properties of EVs 51.

Apart from the above-mentioned common EV isolation techniques based on different sedimentation coefficients, densities or molecular weights, some new techniques for efficiently and specifically obtaining EVs have emerged. Immunoaffinity capture is mainly based on specific binding between specific biomarkers such as surface antibodies of EVs and antibody-recognized ligands immobilized on beads or filters, in which additional elution procedure is required to discard unbound fraction and collect the desired bound EVs. Such method is characterized by high specificity, purity and high cost at the same time 52. Size-exclusion chromatography is a highly reproducible approach based on the different sizes of EVs which exhibit various elution times passing through porous resin particles. It is worth noting that the structural integrity and biological activity of EVs isolated by this method can be preserved while it takes a long run time and a high cost.^50^ Polymer precipitation is also a method that can effectively keep the native state of EVs with the characteristics of easy accessibility and short operation time, in which it takes advantage of highly hydrophilic water-excluding polymers to reduce the solubility of EVs in the mixed solution, then the precipitated EVs can be further separated by low-speed centrifugation 53. However, this method co-precipitates non-EVs contaminants which may affect downstream analysis and quantification of the as-obtained EVs. Recently, a newly-emerged microfluidic technique has attracted tremendous interest in collecting EVs by using parameter differences of the microfluidic channels (*e.g.*, length, diameter, and material), physicochemical or biological variations of the EVs (*e.g.*, immunoaffinity, size, and density), and even additional field forces (*e.g.*, magnetic fields and electrical fields), which typically require low sample consumption and fast processing time. Attractively, the microfluidic technique is more suitable for the quantitative detection of EVs, holding great promise for clinical disease diagnosis due to a handful of starting materials and high sensitivity 54.

Of note, on the basis of the inherent pros and cons of each EV isolation technology, there is currently no specific EV separation technique that is considered to be suitable for all research 55. Therefore, the combined application of two or more separation techniques provides a reasonable strategy for the efficient isolation of EVs, which usually increases the processing complexity and preparation cost, thus affecting downstream analysis of the obtained EVs. In this regard, when choosing a particular combination of techniques, researchers need to carefully consider the intrinsic nature and particular function of the EV samples. Meanwhile, new strategies with favorable reproducibility are needed for the mass production of EV formulations with satisfactory consistency.

## Therapeutic Mechanisms of the Native EVs as Regenerative Entities

**Figure 5:**
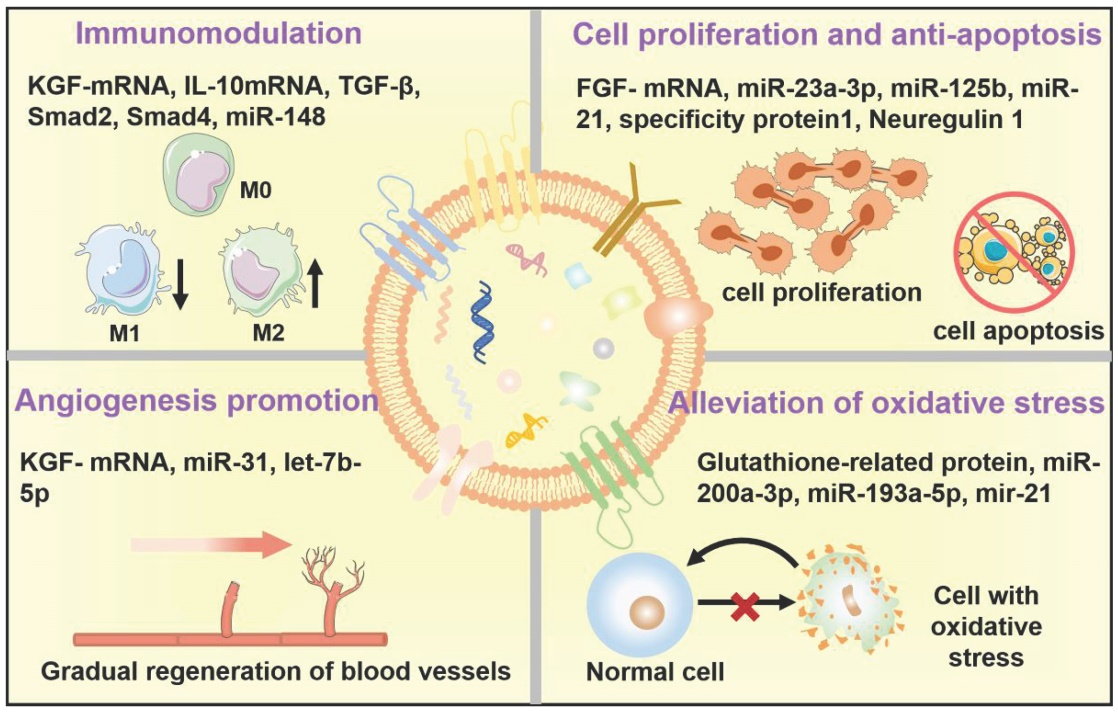
Therapeutic mechanisms of the native EVs as regenerative entities, mainly involve immunomodulation, cell proliferation and anti-apoptosis, angiogenesis promotion, and alleviation of oxidative stress.

As key molecules for intercellular communication, EVs extensively mediate the exchange of information in both physiological and pathological states of cells, which influence the function of recipient cells 63. For example, the release and uptake of EVs may conduce to the progression and metastasis of different diseases 64. Therefore, the therapeutic mechanisms and applications of EVs have been broadly explored. The underlying mechanisms of the therapeutic action in native EVs are attributed to the cell-surface interactions between EVs and the target cells as well as the subsequent transfer of the cargos from EVs to target cells, which will trigger a series of signaling pathways. Several well-studied signaling pathways associated with EVs have been reported, including pAkt/mTOR 65, Erk1/2 66, STAT 67, TGF-β/Smad 68, Efna3 69, and Hedgehog signaling 70. These significant signaling pathways will regulate various physiological functions of the body, including mitigating or eliciting immune responses, reducing inflammation, inhibiting apoptosis, promoting angiogenesis, and minimizing oxidative stress (**Figure 5**), eventually ameliorating the adverse effects of diseases and promoting regenerative functions 11.

### EVs in immunomodulation

Until now, numerous research groups have disclosed the immunomodulatory effects of EVs in various disease models 71,72. In general, EVs can play an immunosuppressive or immunostimulatory role in response to the difference of the active ingredients within EVs as well as the pathological characteristics of diverse diseases 73. In particular, for cancer therapy utilization, research on EVs mainly focus on how to effectively reduce immunosuppression of tumor microenvironments and eventually improve cancer immunotherapy by taking advantage of the immunostimulatory function of EVs. In contrast, for regenerative medicine application, immunosuppression capability stemming from EVs has been subtly harnessed as a crucial therapeutic modality for alleviating inflammation and promoting tissue regeneration. For example, according to the study of Zhu *et al.*, EVs derived from human bone marrow mesenchymal stem cells (BM-MSCs) can effectively alleviate inflammation in a mouse acute lung injury model by down-regulating the expression of macrophage inflammatory protein-2, which may be related to the expression of keratinocyte growth factor (KGF)-mRNA in the injured site 74. It has been well-studied that lots of specific miRNAs highly expressed in EVs have shown crucial immunomodulatory roles, as well as some enriched proteins, such as the contained miR-142-3p, and miR-126-3p can promote dendritic cell maturation and lead to an intrinsic anti-inflammatory outcome of EVs 75, while the contained miR-21a-5p and miR-223 can reduce the expression of inflammatory factors and simultaneously trigger macrophage polarization towards anti-inflammatory M2 phenotype 76,77. And Kim* et al.* have demonstrated that the levels of transforming growth factor (TGF) β1, pentraxin 3, let-7b-5p within EVs greatly affected the immune response in T cell receptor (TCR)- or Toll-like receptor 4 (TLR4)-stimulated splenocytes, and mediating the therapeutic effects of MSC-EVs for the treatment of ocular Sjög-ren's syndrome 33.

### EVs in regulation of cell proliferation and anti-apoptosis

As is well-documented, apoptosis plays a resistance role in tissue regeneration following injury such as ischemia 78. Encouragingly, EVs derived from MSC have been proved to promote tissue regeneration by attenuating apoptosis in various disease models 16,79. Recently, Liu and coworkers demonstrated that exosomes derived from bone-MSCs could efficiently attenuate neuronal cell apoptosis and greatly inhibit the activation of A1 neurotoxic reactive astrocytes, which results in suppressing glial scar formation, alleviating inflammation, promoting axonal regeneration, and eventually improving functional behavioral recovery in spinal cord injury (SCI) rat model 80. Meanwhile, Vesna's team has proved that several growth factors including brain-derived neurotrophic factor (BDNF), fibroblast growth factor-1 (FGF-1), glial cell-derived neurotrophic factor (GDNF), insulin-like growth factor-1 (IGF-1) and nerve growth factor (NGF) within EVs derived from adipose-MSCs could increase the growth of neurite and ultimately enhance sciatic nerve regeneration both *in vitro* and *in vivo* 81. Similarly, EVs derived from human umbilical cord-MSCs could stimulate injured tubular cells to produce a high concentration of hepatocyte growth factor (HGF), which subsequently accelerated tubular cells growth and prevented injured cell apoptosis by activating the signaling of Erk1/2 66.

### EVs in angiogenesis promotion

It has been well-documented that angiogenesis is a crucial process in promoting tissue repair 82,83. Therefore, EV-induced angiogenesis is believed to be considered an important therapeutic mechanism for EV regeneration applications 84. Research has verified that EVs derived from stem cells or endothelial cells containing miR-210, miR-132, and miR-214, could promote cardiac angiogenesis and vascular regeneration in myocardial ischemia 85. For example, Shabbir *et al.* reported that exosomes derived from MSCs can positively activate AKT, Erk, and sata3 signaling pathways in wound healing and induce the expression of growth factors, such as HGF, NGF, IGF-1, and stromal-derived growth factor-1 (SDF1), thereby promoting the proliferation and migration of vascular endothelial cells to form new blood vessels, eventually accelerating skin healing 86. In addition, Wang and coworkers discovered that EVs derived from MSC could also improve angiogenesis *via* miR-210 in the mouse myocardial infarction model, which might be associated with the inhibition of the Efna3 signaling pathways 69. Moreover, Li *et al.* have demonstrated the potential of exosomes derived from macrophages could inhibit inflammation and accelerate the healing of diabetic wounds in the skin defect model rats by synergistically inhibiting the secretion of IL-6 and TNF-α, inducing the proliferation and migration of endothelial cells, as well as promoting angiogenesis of diabetic wounds 87.

### EVs in alleviation of oxidative stress

In addition to the above-mentioned potential therapeutic mechanisms related to native EVs as regenerative entities, EVs can also affect oxidative stress. Overproduction of reactive oxygen species (ROS) and resultant oxidative stress are closely associated with the pathogenesis of various diseases by breaking the redox homeostasis 88-94, which will be an effective target to restore the repair and regeneration capacities. Indeed, a rich variety of research has demonstrated the ROS-scavenging and antioxidative capabilities of EVs 95,96. For example, nuclear-related factor 2 (Nrf2) is a transcription factor that acts as a key regulator in maintaining redox homeostasis in cells 97. Exosomes released from MSC can repair oxidative stress-induced skin injury *via* adaptive regulation of the Nrf2 defense system 98. Moreover, Fafián-Labora *et al.* demonstrated that EVs derived from young human donor fibroblasts exhibited a glutathione-S-transferase activity, which can ameliorate senescence-induced tissue damage of old mice by increasing the old cells' antioxidative capacity, further promoting physical recovery 99.

Collectively, tissue regeneration is a cascading and progressive process, which is a comprehensive result of multiple elements rather than relying on the promotion or suppression of a single pathologic factor. In general, the regenerative performance of EVs is superimposed, which may be involved in immunomodulatory, cell proliferation and anti-apoptosis, tissue angiogenesis, antioxidation, and anti-inflammation at the same time. In this regard, we summarized the regenerative mechanisms of specific components in EVs from the perspective of EVs-containing cargos (miRNA, mRNA, protein) in different disease models (**Table 2**). Among these cargos, miR-21, IL-10 and VEGF-mRNA within EVs have been considered to play a major role in tissue regeneration 100-102. However, the precise mechanisms underlying the therapeutic effects of EVs remain to be fully elucidated for specific diseases in the future.

## Engineering Strategies of the Modified EVs as Regenerative Entities

Besides the native EVs utilized as regenerative entities through multiple therapeutic mechanisms, the modified EVs can also be applied for regenerative applications by using different engineering strategies which mainly focus on cargo loading and membrane modification. As such, the modified EVs show enhanced therapeutic efficiency and additional targeted function over the native EVs. Particularly, a rich variety of exogenous therapeutic molecules, such as small molecular drugs, nucleic acid, and proteins are loaded into the interior of EVs or embedded into the EV membrane by various methods before or after EV secretion. In general, the reported engineering strategies of EVs can be roughly divided into two broad categories, namely endogenous engineering strategy and exogenous engineering strategy. The endogenous approaches refer to the modification of the parent cells before EV isolation, mainly including genetic, physical and chemical manipulations, while the exogenous approaches briefly summarized indicate post-isolation-functionalization of the nanoscale-EVs, mainly including incubation, electroporation, extrusion, sonication, freeze/thaw cycles and direct EV membrane modifications by utilizing covalent or non-covalent interaction.

### Endogenous engineering strategy

#### Genetic engineering strategy

Surface modification and additional functionalization of EVs can be achieved by genetically manipulating the protein biosynthesis process of target cells, in which plasmids and viral vectors are commonly served as molecular biology tools for transgene expression 27,118. Attractively, genetic engineering strategies may endow the modified EVs with targeting, tracking and additional therapeutic properties by enriching some specific biomacromolecules such as proteins 119 and RNA (microRNA or small interfering RNA) 120 in parent cells. For example, Li *et al.* have demonstrated that transfecting MSCs with miR-133b gene resulted in an accumulation of nearly 2.5-fold higher levels of miR-133b in EVs compared to the control group, which performed a better functional recovery performance in the SCI model 121. Besides, Casella's team have empowered EVs derived from the murine BV-2 microglial cells with remarkable anti-inflammatory effect by transfecting the source cells with a plasmid coding for anti-inflammatory cytokine IL-4. Such genetically modified EVs with overexpression of the “eat me” signal Lactadherin (Mfg-e8) on the surface could actively target phagocytes of the brain, leading to substantially reduced neuroinflammation in the mouse model of multiple sclerosis as well as autoimmune encephalomyelitis 122.

#### Chemical engineering strategy

Besides genetic engineering, EV donor cells can also be modified by a chemical engineering strategy, which commonly combines metabolic engineering and clicks chemistry 123. Intriguingly, these covalent chemical reactions make the EV functionalization more stable when compared to the noncovalent engineering methods, especially in the long blood circulation, eventually endowing the modified EVs with new surface compositions and additional functions 124. For example, Lim and coworkers introduced exogenous azide groups (-N_3_) on the surface of EV-secreting donor cells *via* metabolic glycoengineering by metabolic glycoengineering (MGE) using unnatural sialic acid tetraacetylated *N*-azidoacetyl-D-mannosamine (Ac_4_ManNAz). Combined with the bioorthogonal copper-free click chemistry, dibenzocyclooctyne (DBCO)-terminated hyaluronic acid was covalently edited on the resultant EV surface to specifically target the CD44-overexpressing cells, which enables a prolonged blood circulation and favorable targeting ability of the modified EVs in both rheumatoid arthritis and tumor mouse models 125. In brief, this facile and safe surface-engineering approach can introduce a wealth of functional moieties onto EVs without variation in their protein expression.

#### Physical engineering strategy

A final endogenous strategy for straightforward EV functionalization is the physical engineering method where the content of specific cargo in EVs could be changed by direct physical force to the donor cells or regulation of cell culture conditions. The common physical methods include incubation, electroporation, and extrusion 126. For example, by changing the culture environment of the donor cells and giving certain stimulation to the cells, it is possible to change the amount of EV released or the type and content of therapeutic factors, thereby optimizing its treatment efficiency. Very recently, Guo's group engineered different tissues respectively seeded with MSCs, human dental pulp stem cells and skeletal muscle cells in a 3D culture and then placed the engineered tissues in bioreactors for two types of mechanical stimulation including flow stimulation or mechanical stretching, thus significantly boosting the EV production yield mediated by Yes-associated protein mechanosensitivity as well as optimizing their functional performance for clinical applications 127.

### Exogenous engineering strategy

#### Co-incubation

Co-incubation by directly co-incubating EVs with various chemical compounds under different conditions, is a simple and inexpensive EV engineering strategy that will not destroy the integrity of the EV membranes and show little effect on their natural functions. Although co-incubation is widely used in EV research, this approach is more suitable for hydrophobic compounds, such as small molecule drugs and specific RNA 22,128. Up to now, extensive research has demonstrated that chemotherapeutic drugs, such as curcumin, doxorubicin, and paclitaxel could be successfully loaded into EVs *via* co-incubation at room temperature 22,129. The loading efficiency mainly depends on the hydrophobicity of the cargo, incubation periods, and other operational details, all of which can influence the interaction between the encapsulated cargo and the lipid membrane of EVs.

#### Electroporation

Electroporation is the most promising strategy for exogenous cargo loading into EVs, in which a high-intensity short-duration voltage-current disturbs the phospholipid bilayer of the EVs and creates small-transit pores on the membrane of EVs. Thus, small molecule drugs or nucleotides can subsequently diffuse into the interior of EVs. The integrity of the membrane is then recovered automatically. Specifically, the electroporation approach is widely used for loading different nucleotides (such as miRNAs, siRNA, antisense oligonucleotides, and plasmids) 130,131 into EVs because these biomacromolecules with large sizes hardly spontaneously diffuse into the EVs 132. Although electroporation has been utilized in all types of EVs and is able to incorporate large compounds, it may affect the zeta potential and colloidal stability of EVs, thus causing RNA aggregation 27. To address this issue, Kooijmans and coworkers reported that EVs aggregation could be dramatically reduced by the addition of ethylene diamine tetraacetic acid before electroporation 133.

#### Sonication

Apart from co-incubation and electroporation, sonication is the third most wildly used exogenous engineering strategy for EV modification. In this method, the mechanical shear force produced by the sonicator probe breaks the membrane integrity of the EVs and promotes the exogenous cargo to diffuse into the EVs during membrane deformation. There are studies demonstrating that the loading efficiency of sonication is much higher than that of co-incubation and electroporation 129. However, this method has the limits in that some cargos may adhere to the outer layer of the films during sonication, thus influencing the release property of the encapsulated cargos 132. Moreover, the mechanical force produced by sonication may affect the integrity of the EV membrane, which will influence the therapeutic activity of the modified EVs.

#### Mechanical extrusion

Mechanical extrusion can be used to encapsulate various exogenous cargos particularly synthetic nanoparticles (NPs) into EVs by extruding the mixture of the cargos and EVs through a syringe-based mini-extruder with polycarbonate membranes of pore size from 200 nm to 400 nm. Briefly, mechanical extrusion deforms the membrane of EVs, further promoting EVs vigorously mixed with the cargo. Currently, the mechanical extrusion method has always been applied to facilitate the encapsulation of synthetic NPs such as IONPs, AuNPs 134. Notably, this method can especially endow the modified EVs with additional therapeutic effects 135, whereas continuous mechanical extrusion may change the membrane stability of EVs, causing the engineered EVs to exert unpredictable side effects 136.

#### Freeze/thaw cycles

Freeze/thaw cycles are a relatively simple but not very commonly used approach for loading different cargos in EV such as chemotherapeutic drugs and enzymes 135,137, in which ice crystals within lipid bilayer membranes will be temporarily formed and subsequently removed as water molecules during freeze-thaw cycles, resulting in disrupting EV membranes to encapsulate exogenous cargos. In a typical freeze/thaw procedure, cargos are firstly incubated with EVs at room temperature for a given time, then rapidly frozen at -80 °C or with liquid nitrogen, finally thawed at room temperature for at least three cycles 138. Although this method avoids contamination of the EV membranes with harmful chemicals, it also meets the challenge of EV aggregation trend and the resultant large particle size of the modified EVs 139.

#### Direct EV membrane modification

In addition to the chemical engineering strategy towards the source cell membrane prior to EV isolation, direct EV membrane modification after EV secretion is another important exogenous approach for EV functionalization *via* either covalent or noncovalent methods 140. Generally, the former method applies bioconjugation, amidation, aldehyde amine condensation, and click chemistry to link molecules on EV surface by chemical bonds, while the latter approach modifies the EV membranes by hydrophobic insertion, receptor-ligand binding, fusion, and multivalent electrostatic interactions 27,141-143. Of note, even through direct EV membrane modification efficiently endows the modified EV with additional functions, it is still unknown whether membrane modification would weaken EVs' endogenous carrier capacity and the long-term biocompatibility, stability, or safety of the engineered EVs remain to be clarified.

In summary, proper engineering methods will result in optimal cargo loading or superb conjugating efficiency in modified EVs and accordingly enhance their targeting capabilities and therapeutic efficacies. The principle, advantages, disadvantages, and applicable molecular types of different engineering strategies for modification of the EVs for biomedicine have been summarized in **Table 3.** To achieve the most satisfying surface modification and additional functionalization outcomes of the modified EVs, several crucial factors should be considered before the selection of the most suitable EV engineering method, such as the type of cargo being engineered, different EV sources, the purity of the modified EVs, as well as the structural integrity and intrinsic bioactivity of the EV itself.

## Tissue Regeneration Applications of EVs on Various Organs

In light of the above introduction on native and modified EVs, currently, there are two main strategies for the utilization of EVs in regenerative medicine. On one hand, native or natural EVs produced from certain source cells such as MSCs or immune cells can be directly served as potential candidates for regeneration therapies. On the other hand, outside of the inherent therapeutic effects, EV itself can be simultaneously acted as a delivery carrier through engineering various therapeutic agents in EVs to achieve a cascading progressive regenerative effect. Herein, we will briefly review the therapeutic application of different EVs on various organs in regenerative medicine, based on the strategies mentioned above.

### Brain

**Figure 6:**
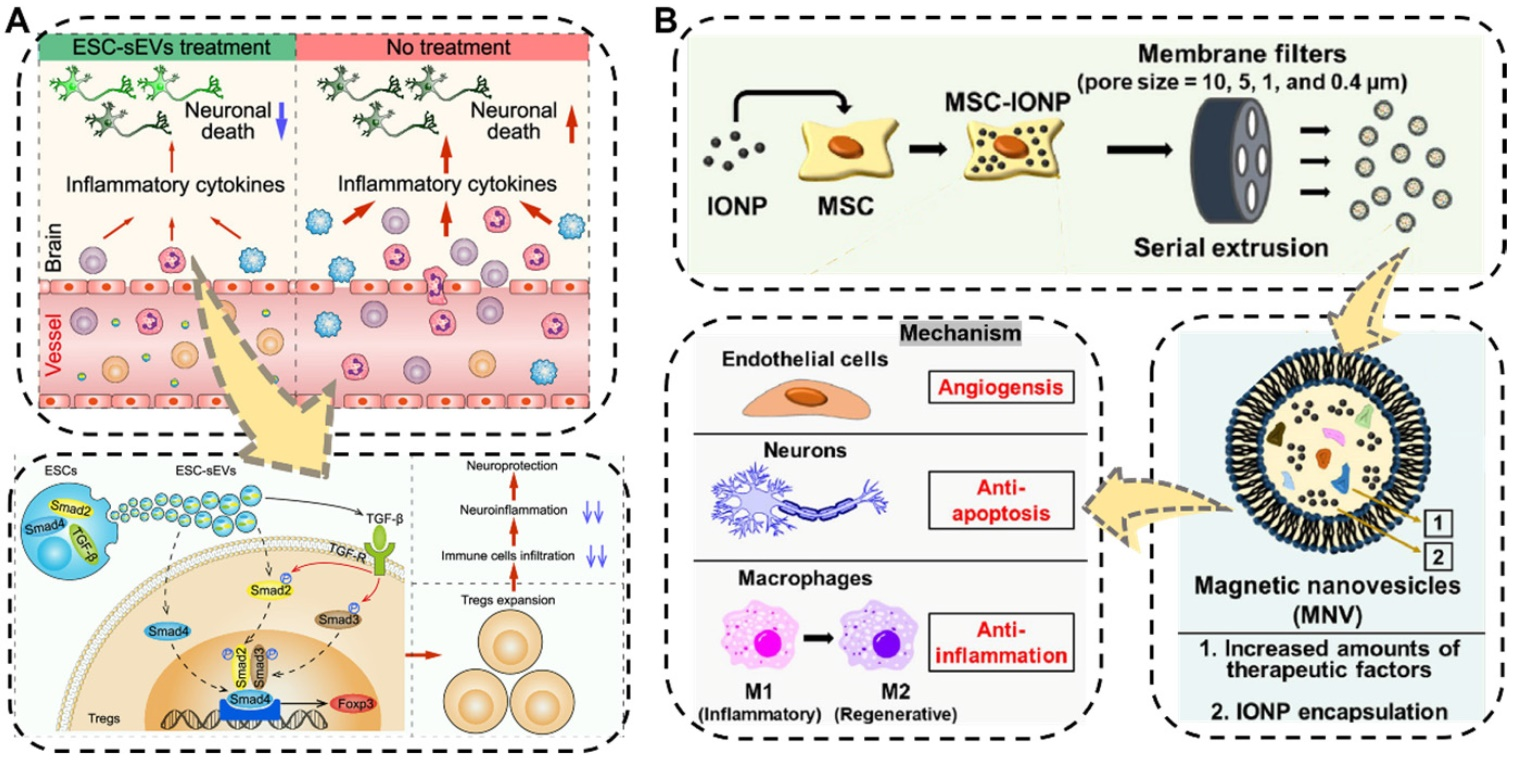
** (A)** Schematic illustration of the ESC-sEVs to induce Treg expansion and alleviate neuroinflammation against ischemic stroke. Reproduced with permission 162. Copyright 2021, American Chemical Society. **(B)** Schematic illustration of the fabrication and multiple therapeutic benefits of the MSC-derived magnetic nanovesicles (MNV) to treat ischemic stroke. Reproduced with permission 165. Copyright 2020, Elsevier.

The brain is one of the most important and most complex organs, serving as the center of the nervous system in the human body. Neurological impairments caused by traumatic brain injury (TBI) and different types of neurodegenerative diseases such as ischemia stroke, Alzheimer's disease, and Parkinson's disease in brain organs, are the major reasons for the long-term physical disability of adults, especially the increasing aged people, posing a considerable threat to public health 158,159. Even worse, the treatment performance of traditional pharmacotherapy towards brain diseases has often been substantially compromised due to the presence of the blood-brain barrier, which makes it difficult for drugs to reach the brain lesions. Hence, the development of new therapeutic regimens capable of crossing BBB is strongly needed. Fortunately, EVs showing BBB-crossing ability which is similar to their parental cells, have been recently applied as ideal potential therapeutics for nerve regeneration and central nervous system repair 141,160,161. For example, very recently, Xia *et al.* proved the neurological recovery function of the embryonic stem cell derived small EVs (ESC-sEVs) which were collected by the classical differential centrifugation method in ischemic stroke mouse models (**Figure 6A**) 162. The as-obtained ESC-sEVs showed significant alleviation of neuroinflammation and obvious decrease of the peripheral immune cells infiltration by promoting Treg expansion *via* the TGF-β/Smad signaling pathway, which resulted in reduced neuronal death as well as nerve regeneration after ischemic stroke. This study suggested that the nature EVs could be utilized as a powerful therapeutic tool for brain injuries and other autoimmune diseases.

In addition to being widely used as native therapeutic agents for neuroregeneration, EVs have also been engineered to transport various cargo for neurological repair 163,164. Kim's group designed a biocompatible magnetic nanovesicle (MNV) by a serial extrusion of iron oxide nanoparticles (IONP)-loaded MSC to greatly enhance the therapeutic outcome against ischemic stroke (**Figure 6B**) 165. On one hand, the MSC nanovesicle itself exhibited multifaceted intrinsic therapeutic benefits including angiogenesis, anti-apoptosis, and anti-inflammation. Moreover, the incorporated IONP would not only further considerably upregulate the expressions of therapeutic growth factors in the MSC but also endow the MNV with brain infarcted lesion targeting capability under an external magnetic field. By means of these advantages, the as-fabricated MNV ultimately ameliorated the neuronal damage in the ischemic lesion and displayed remarkable therapeutic outcomes in the treatment of ischemic stroke.

### Heart

The human heart is a primary organ in the circulatory system, playing a central role in delivering oxygen and nutrients to the tissues through the pumped blood. In recent decades, cardiovascular diseases (CVD) are the top leading cause of death all over the world according to the World Health Organization, causing both health and economic burdens for patients and families 166,167. Especially, myocardial infarction, one of the representative CVD and the leading causes of mortality worldwide, is caused by acute or persistent ischemia and hypoxia of coronary artery, resulting in subsequent cell apoptosis and cardiac dysfunction. With the purpose of restoring cardiomyocytes, stem cell-based treatments have been extensively implemented for MI in the past years 168-170. More attractively, numerous preclinical studies have found that the MSC-EVs have the similar myocardial regeneration functions with their donor cells *via* proliferation of the cardiomyocytes, inhibition of cardiac cell apoptosis, pro-angiogenesis, and reduction of infarct size 171. Li and co-workers demonstrated that EVs from hypoxia-preconditioned MSCs can promote cardiac repair by improving cardiac function, enhancing vascular density, and reducing infarct size *via* the enriched miR-486-5p in mice and nonhuman primate MI models 172. Similarly, EVs derived from Krüppel-Like Factor 2-overexpressing endothelial cells also exhibited the cardioprotective effects, which could improve heart injury and relieve inflammation level in heart by restraining the Ly6C^high^ monocyte recruitment through the contained miRNA-24-3p 173.

**Figure 7:**
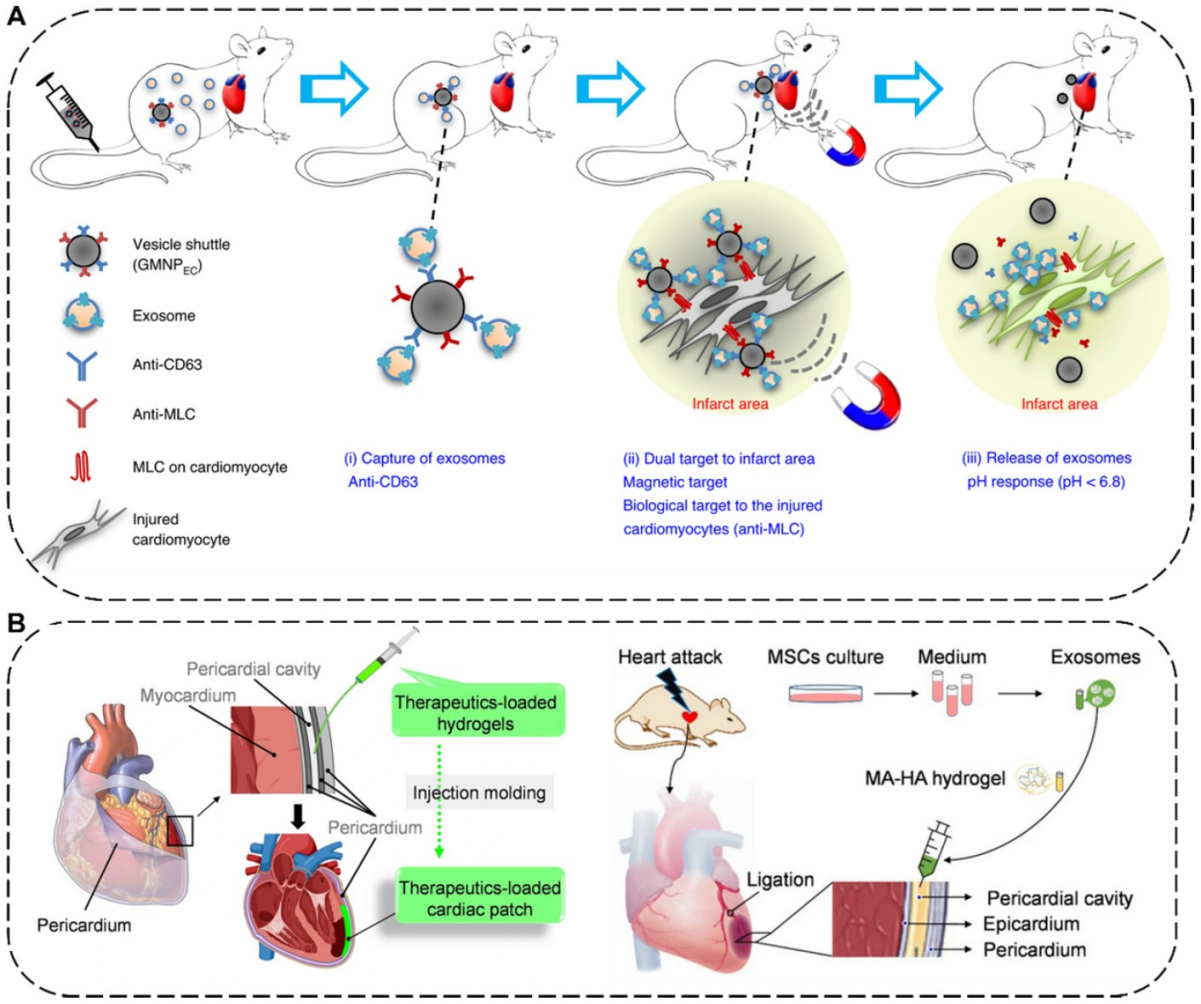
** (A)** Schematic illustration of the grafted magnetic nanoparticle (GMNP) as the *in vivo* vesicle shuttle to selectively capture, transport and release circulating exosomes 176. Copyright 2020, Springer Nature. **(B)** Schematic illustration of *in situ* cardiac patch formation after intrapericardial delivery of biocompatible hydrogels containing exosomes for MI therapy 174. Copyright 2021, Springer Nature.

The delivery methods of EVs for MI therapy include intravenous, intramyocardial, intracoronary, and intrapericardial injections 174. Among them, intravenous injection is the most preferred approach for EV delivery due to its satisfactory safety and superior convenience 175. For example, to manipulate the systemic biodistribution and local concentration of endogenous EVs for MI therapy, Liu *et al*. have designed a dually-targetable grafted magnetic nanoparticle (GMNP) as the *in vivo* vesicle shuttle to selectively capture, transport and release circulating therapeutic exosomes with proangiogenic and cardiac repair functions by intravenous injection for ameliorating angiogenesis and heart function (**Figure 7A**). Particularly, the GMNP surface engineered two types of antibody (GMNP_EC_) *via* an acid-cleavable hydrazone bonds, in which one antibody anti-CD63 can bind to CD63 antigens on the surface marker of EVs and the other antibody anti-MLC can target to the myosin-light-chain surface markers on the injured cardiomyocytes. By taking advantages of an external magnetic field and the conjugated anti-MLC antibody, the endogenous circulating therapeutic exosomes captured by GMNP_EC_ would be actively accumulated and responsively released in the acidic infarcted heart areas. In both rabbit and rat MI models, the released exosomes in injured cardiac tissue significantly led to reduction in infarct size, promotion of angiogenesis, and improvement of heart functions 176. In addition to injection, placing a cardiac patch on the surface of the heart is another effective way to deliver therapeutics to the heart 177. Very recently, Cheng's group has combined* in situ* cardiac patch formation with intrapericardial injection of biocompatible hydrogels containing EVs to achieve minimally invasive delivery of therapeutics into the pericardial cavity for cardiac repair (**Figure 7B**). The intrapericardial injection-based cardiac patch showed robust cardiovascular repair performance and improved cardiac functions in both mouse MI model and a clinically relevant porcine model 174.

### Bone and cartilage

**Figure 8:**
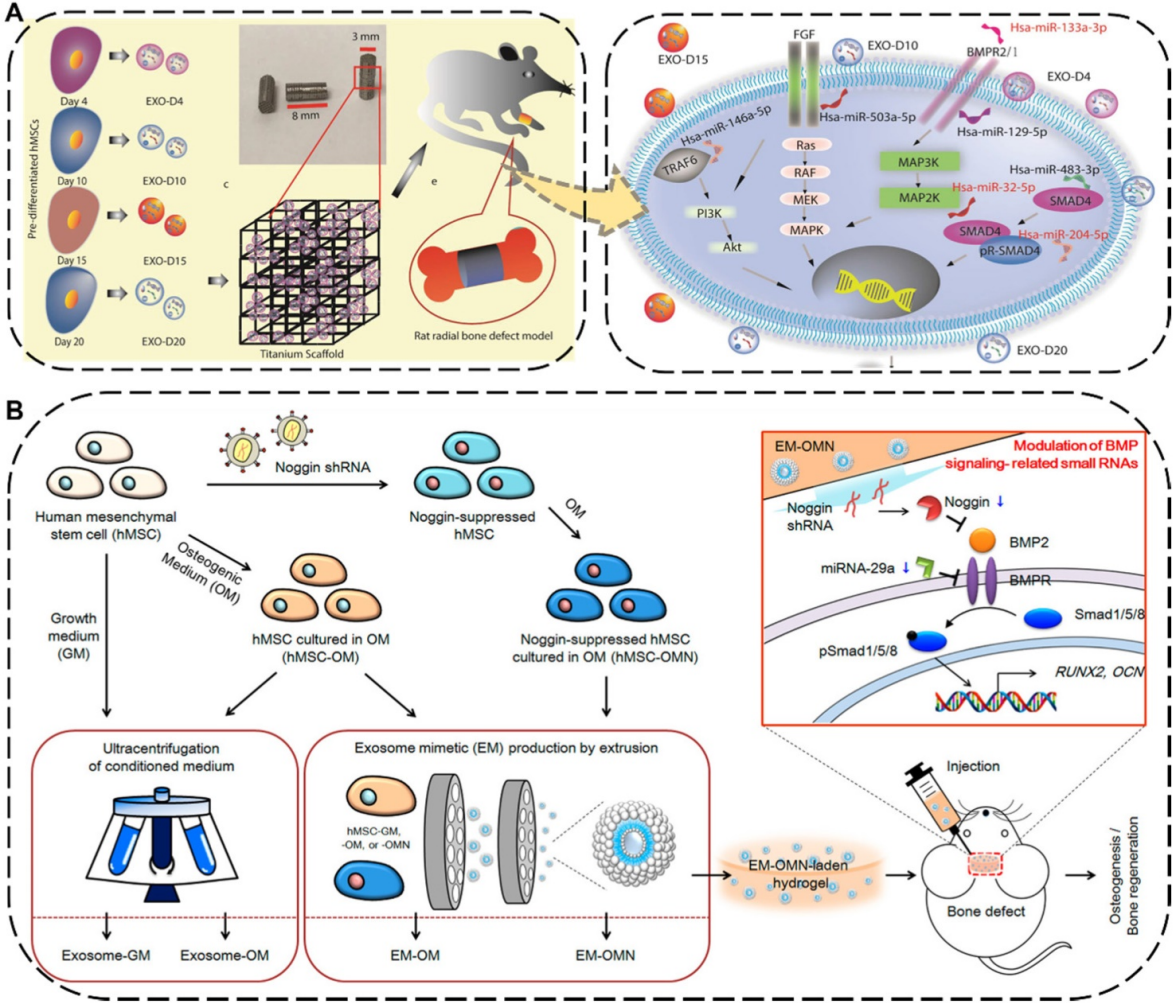
** (A)** Schematic diagram and possible mechanisms of the hMSCs-derived exosomes decorated 3D printed titanium alloy scaffolds for bone regeneration. Reproduced with permission 184. Copyright 2020, WILEY-VCH. **(B)** Schematic illustration and mechanism of the hMSCs-EMs for calvarial bone regenerative. Reproduced with permission 185. Copyright 2020, American Chemical Society.

The injuries and defects of bone or cartilage caused by trauma, degenerative diseases, aging, and other factors are extremely common in daily life, such as fractures, bone defects and osteonecrosis 178. As a common orthopedic symptom and typical complication, the bone defect can be caused by various diseases such as bone infections, bone tumors, skeletal abnormalities, fractures, osteoporosis, and trauma 179,180. Traditionally treating with transplants against bone defects has limitations of donor source and immune rejection 181. To overcome this intractable dilemma, the implantation of EV-based cell-free scaffolds has emerged as a new promising strategy for bone regeneration therapy, where the embedded EVs can directly program macrophages to an M2 phenotype, promote osteoblasts function, facilitate mineralization, as well as indirectly boost vascularization, eventually resulting in desirable bone regeneration performance 182,183. For instance, Zhai *et al.* used exosomes derived from human mesenchymal stem cells (hMSCs) which pre-differentiated for different durations ranging from 4 to 20 days to decorate 3D-printed titanium alloy scaffolds (Ti-scaffolds) for bone regeneration (**Figure 8A**) 184. Specifically, osteogenic exosomes pre-differentiated for 10 and 15 days (termed EXO-D10 and EXO-D15) would be obtained. Afterward, such exosome-coated Ti-scaffolds efficiently induced the *in vitro* osteogenic differentiation of hMSCs and subsequently regenerated bone tissue in the rat radial bone defect model by simultaneously upregulating the osteogenic miRNAs and downregulating the anti-osteogenic miRNAs within the osteogenic exosomes to trigger the PI3K/Akt and MAPK signal pathways.

To further improve the regenerative ability and production yield of the hMSCs-derived exosomes, Fan *et al.* applied an extrusion approach to amass the exosome mimetics (EMs) from hMSCs for skeletal repair (**Figure 8B**) 185. The EMs secreted from the noggin-knockdown hMSCs by transduction of noggin shRNA (termed EM-OMN) were then encapsulated into chitosan hydrogel, which exhibited robust bone healing capability in rodent calvarial defect models *via* inhibition of miR-29a to enhanced osteogenesis. In addition to being widely used for bone regeneration, numerous studies have recently reported that EVs can also be effective in promoting the cartilage repair and regeneration. Zhang *et al.* demonstrated that exosomes derived from MSCs could display an efficient osteochondral regeneration outcome by promoting the proliferation of chondrocytes, enhancing matrix synthesis, and modulating a regenerative immune phenotype *via* exosomal CD73-activated AKT and ERK signal pathways 186.

### Liver and kidney

**Figure 9:**
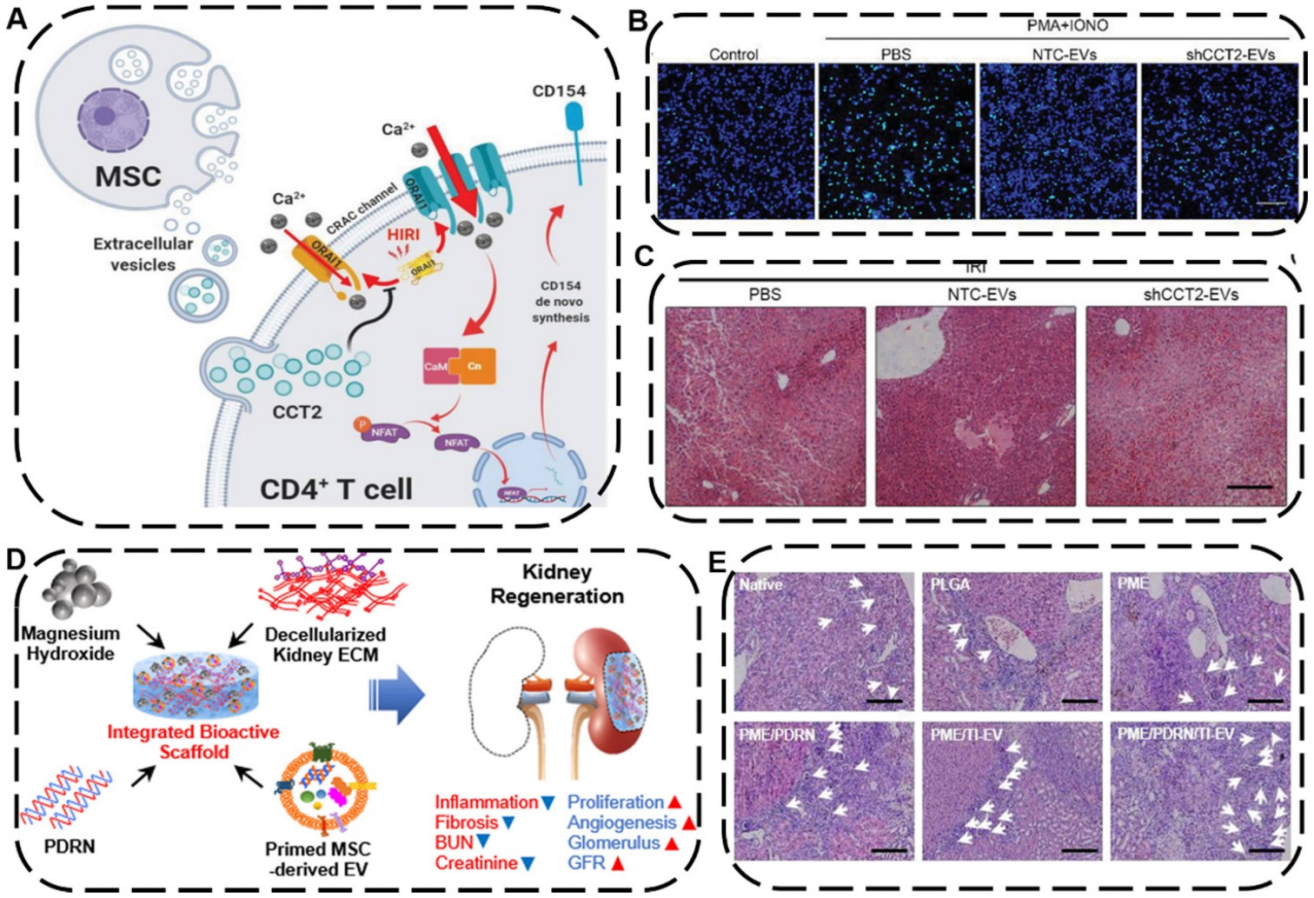
** (A)** Schematic mechanism of HUC-MSC-EVs with hepatoprotective and immunoregulative outcomes. **(B)** The green fluorescence represented the level of Ca^2+^ influx in CD4+ T cells in different groups. Scale bars: 60 µm. **(C)** H&E staining of liver sections after different treatments. Scale bars: 200 µm. Reproduced with permission 191. Copyright 2020, Wiley-VCH. **(D)** Schematic diagram and possible mechanisms of PDRN/TI-EVs fabrication for kidney tissue regeneration. **(E)** Representative H&E staining of regenerated glomeruli. Scale bars: 100 µm. Reproduced with permission 189. Copyright 2021, American Chemical Society.

The liver and the kidney are two of the most pivotal organs in human body carrying out various essential functions, including detoxification, excretion of waste, protein synthesis, electrolyte balance, and metabolites production. Currently, acute liver failure has been considered as one of the leading causes of death globally 187, while acute kidney injury also associated with high mortality often raises the risk of chronic kidney disease (CKD) and end-stage renal diseases 188. Liver or kidney transplantation is the most effective and curative therapy for the above-mentioned diseases so far, which is substantially limited by high costs, lack of donors, and graft rejections 189. Recently, as an alternative to organ transplantation, EV-based cell-free therapy has been shown to be greatly effective on treating liver or kidney failures 190. For instance, Zheng *et al.* demonstrated that EVs derived from HUC-MSCs can protect liver injury in mice ischemia/reperfusion injury (IRI) model through downregulating CD154 expression on intrahepatic CD4+T cells because the CD154 highly expressed CD4+T cells would trigger the inflammatory response in liver and deteriorate liver IRI. Further experiments proved that Chaperonin Containing TCP1 Subunit 2 (CCT2) within HUC-MSCs-EVs could modulate the calcium channels to reduce the CD154 synthesis on CD4+ T cells, leading to a robust therapeutic potential of UC-MSC-EVs in alleviating IRI in liver (**Figure 9A-C**) 191.

To achieve a combined tissue regeneration effect for CKD therapy, Ko's group developed a multifunctional PME/PDRN/TI-EV scaffold consisting of poly (lactic-co-glycolic acid) (P), magnesium hydroxide (M), and porcine kidney extracellular matrix (E), which further engineered with therapeutic components polydeoxyribonucleotide (PDRN) as well as EVs derived from tumor necrosis factor-α (TNF-α) and interferon-γ (IFN-γ)-loaded MSCs (TI-EVs) (**Figure 9D**) 189. Intriguingly, the integration of PDRN and TI-EVs displayed a remarkable synergistic outcome in the regeneration and restoration of a functional kidney tissue by promoting cell proliferation, boosting angiogenesis, and alleviating fibrosis as well as inflammatory response, ultimately making the as-fabricated bioactive PME/PDRN/TI-EV scaffold an efficient tissue regenerative platform in a partial nephrectomy mouse model (**Figure 9E**). Based on the above examples, both the native EVs themselves and EVs-engineered scaffolds can be utilized as an advanced regenerative entity for liver or kidney tissue regeneration.

### Skin

**Figure 10:**
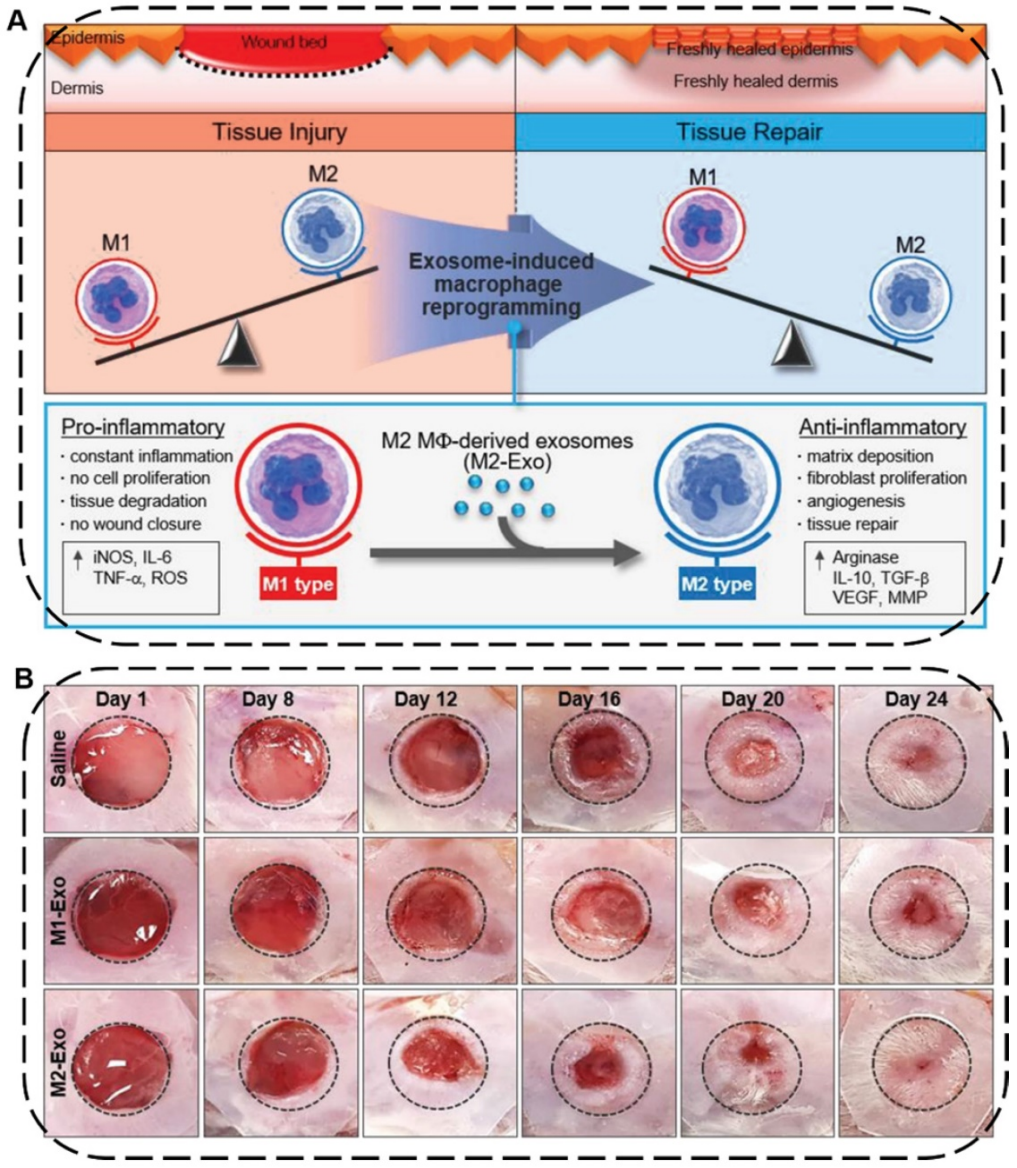
** (A)** Schematic illustration of macrophage reprogramming induced by exosomes derived from M1- and M2-type macrophages for tissue injury or repair. **(B)** Representative images of wound closure *in vivo* after local injection of saline, M1-Exo, and M2-Exo. Reproduced with permission 194. Copyright 2019, WILEY-VCH.

Skin injuries are common acute or chronic epithelial tissue injuries often caused by trauma, burns or diabetes. Cutaneous wound healing is a highly programmed process generally involving four steps: hemostasis, inflammation, proliferation, and remodeling phases 192. During this process, macrophages play an important role in wound healing where the pro-inflammatory M1-type macrophages can induce constant inflammation and tissue degradation as well as inhibit cell proliferation and wound closure while the anti-inflammatory M2-type macrophages can effectively boost fibroblast migration, angiogenesis, and tissue repair 193. In this regard, EVs secreted from macrophages or stem cells can be used to facilitate wound healing and skin tissue regeneration by regulating immune responses, polarizing macrophages from M1 to M2, and promoting the proliferation of skin cells and angiogenesis. For example, Kim and coworkers proposed an exosome-guided phenotypic transformation of macrophages from pro-inflammatory M1 to anti-inflammatory M2 phenotype for cutaneous wound repair (**Figure 10A**) 194. Importantly, the M2-type macrophages derived exosomes (M2-Exo) not only directly reprogrammed M1 to M2-type macrophages showing high tissue regenerative capability, but also released various luminal cytokines and growth factors including interleukin 4 (IL4), C-X-C motif chemokine ligand 12 (CXCL12), and basic FGF to promote angiogenesis and re-epithelialization through the paracrine secretion, synergistically accelerating cutaneous wound healing and repair in mouse skin wound model (**Figure 10B**).

## EVs Formulations for Regenerative Medicine in Clinical Trials

In recent few decades, with more in-depth research on the biogenesis and functions of EVs, either nature or modified EVs have become promising bioactive nanotherapeutics for the diagnosis and treatment of various diseases 11,195. In 2005, Zitvogel and coworkers conducted the Phase I clinical trials of the tumor-therapeutic EVs derived from the autonomic dendritic cells of patients with malignant melanoma and then confirmed their clinical safety, which was the first clinical trial on EVs 23. Currently, there are mounting clinical trials on EVs used as biomarkers, therapeutic agents or drug carriers for cancer treatment 25,196-198. However, clinical research on tissue repair and regeneration applications of EVs is still being developed, basically in phases I and II stages, which mainly focus on evaluating the safety and effectiveness of EVs. For example, one study aimed to evaluate the effect of autologous EVs-rich plasma on patients with cutaneous wound healing. The participants have been treated with specific plasma every day for 28 days (https://www.clinicaltrials.gov/ct2/, NCT02565264). Another study aims at assessing the safety and efficacy of AGLE-102 in the treatment of lesions in subjects with epidermolysis bullosa, where the AGLE-102 is an allogeneic EV product derived from MSCs of a healthy person. This study is expected to begin in April 2022 (NCT04173650).

Of note, since the novel coronavirus (COVID-19) outbreak began at the end of 2019, the discovery of fatal cytokine storms in patients with moderate to severe COVID-19 diseases has spawned some clinical trials to use EV products for COVID-19 treatment because the EVs can regulate immunity, restore oxygenation, and suppress inflammation. Recently, a clinical trial of MSC-derived exosomes has been carried out to reduce inflammation in critically ill patients with COVID-19, in which the MSC-derived exosomes can greatly inhibit the pulmonary fibrosis pathways (NCT05191381). Actually, as early as in 2020, Dinh *et al.* have also found that lung spheroid cell-derived exosome can alleviate the symptoms of pulmonary fibrosis caused by bleomycin and silica through reestablishing normal alveolar structure, reducing collagen accumulation, and decreasing myofibroblast proliferation, ultimately promoting lung regeneration 199. In addition, during April 2020, 24 moderate-to-severe acute patients of COVID-19 received ExoFlo™ treatment, in which the ExoFlo™ was a BM-MSCs derived EV product. Both safety and efficacy of the ExoFlo™ from day 1 to 14 post-treatment were evaluated. Clinal results revealed that patients' oxygenation improved while neutrophil counts and acute phase reactants declined, suggesting that the EV product is a promising therapeutic candidate for severe COVID-19 (NCT04493242)^200^. Overall, all these preliminary results from clinical trials encourage further development of EVs formulations as a potential candidate for regenerative medicine.

## Future Challenges and Perspectives

**Figure 11:**
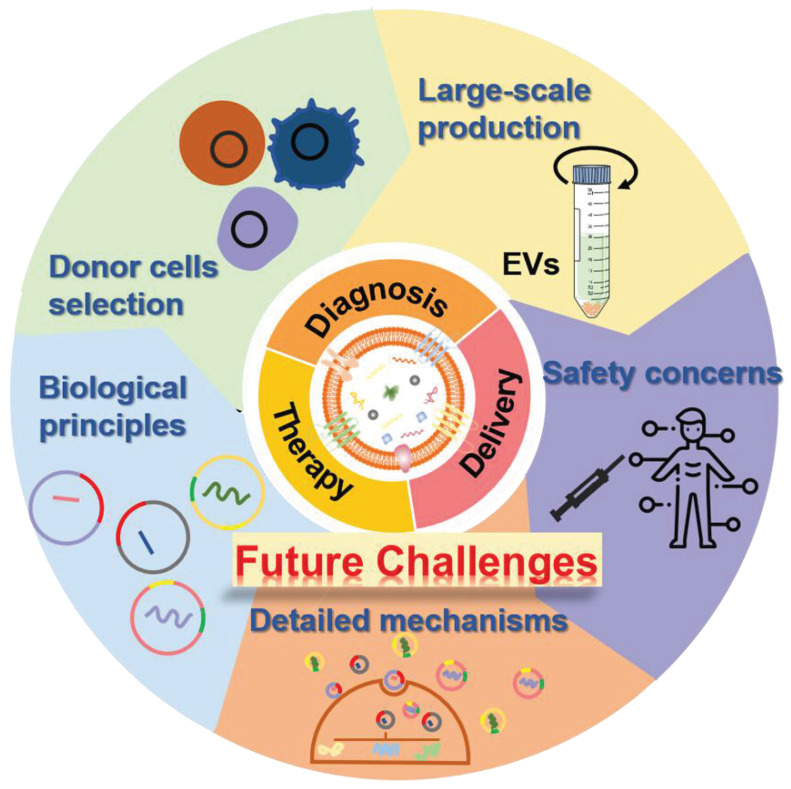
Future challenges of EVs as bioactive nanotherapeutics for biomedical application and clinical translation.

The past few decades have shown that the natural cell-derived EVs with fascinating merits including inherent biological functions, low immunogenicity, desirable biocompatibility, as well as ability to realize disease-specific drug delivery, play significant roles in a wide range of physiological or pathological processes as well as a rich variety of diseases treatments, especially in regenerative medicine, which not only affording very powerful tools for diagnostics and therapeutics but also providing new opportunities for clinical translation of the nanotherapeutics. Hence, a large amount of native or engineered EVs have been extensively studied and fabricated for the treatment of various diseases. Although several achievements have been obtained in preclinical trials of EV-based nanotherapeutics, there are still several ongoing scientific challenges and key technical issues that need to be addressed before their further widespread applications and practical clinical utilization (**Figure 11**).

### Donor cell selection and EV heterogeneity

There is increasing evidence that EVs from different donor cells show distinct physicochemical indexes (*e.g.*, structural/compositional variation and different tissue biodistribution patterns), thus leading to their distinct biological functions and subsequent therapeutic outcomes. Particularly, some certain subpopulations with unique morphological and physicochemical features may be more suitable than other subpopulations for specific biomedical applications. Besides, cell passage number as well as cell culture environment can also affect the actual functions of EVs, which substantially determines whether EVs perform a therapeutic or pathogenic effect. For example, EVs generated from early passaged MSCs may have better therapeutic efficacy than EVs generated from late passaged MSCs, while EVs generated from 3D cultured MSCs may be more effective than 2D EVs 33. Therefore, the selection of appropriate EV donor cells and subtypes with favorable biological behaviors is of great importance for personalized disease treatment, especially for accurate regenerative medicine applications. In addition, EV heterogeneity referring to the inconsistency of size, component content, protein type, specific function, and other biophysics/ biochemical properties can be found in the production of EVs, even isolated from the same donor cells, which would also impact the ultimate therapeutic performances of EVs. In this regard, the questions of which type of donor cell is mostly suitable for EVs generation, how to deal with the differentiation in EVs production under the same or different cell culture conditions, and how to reduce the variability of EVs due to EV component plasticity during their preparation process should be further critically investigated.

### Comprehensive insights on the biological principles of EVs

Although much information on the biological characteristics of EVs including their biogenesis, general subpopulation, cargo sorting, and basic biological behaviors have been revealed to some extent, the current understanding on EV biology is still preliminary and partial. More comprehensive and in-depth insights on the biological principles of EVs should be required. For example, further elucidation on the detailed and accurate process of EVs formation could facilitate the subsequent preparation, isolation, and purification of the objective EVs with satisfactory controllability. Furthermore, a thorough but deeper investigation dedicated towards *in vivo* interactions and fates of EVs including blood circulation, biodistribution, and elimination in a complex biological milieu as well as the following intracellular fates after internalization will give a more profound understanding on the pharmacokinetic and pharmacodynamic performance of the EVs, which will be beneficial for the rational design of an EV-based nanotherapeutic as targeted strategies for various biomedical applications.

### Detailed mechanisms in tissue repair and regeneration

Despite recent extensive studies on various therapeutic mechanisms of native or engineered EVs for different diseases, especially for cancer and inflammatory diseases, the potential roles of specific functional units from EVs which endow the EVs with functions of tissue repair and regeneration have not been fully clarified. As previously mentioned, we introduced several currently proposed therapeutic mechanisms of the native EVs as regenerative entities, such as immunomodulation, regulation of cell proliferation and angiogenesis, as well as anti-apoptosis. Nevertheless, the detailed molecular mechanisms and corresponding target signal pathways remain elusive and require to be further elucidation, which are of fundamental importance in understating the therapeutic actions in regenerative medicine and broadening the biomedical applications of EVs.

### Undesirable biological effects and safety concerns

One of the promising advantages of EVs over conventional stem cell therapy that we have emphasized earlier is their enhanced biocompatibility and biosafety. However, the whole components of EVs as well as the bioactivities of nucleic acids and proteins or other unknown molecules within EVs are still not completely clear, which would probably induce unpredictable or undesirable biological effects, thus, unfortunately leading to potential safety concerns of EVs for their clinical application. On the other hand, EV heterogeneity mentioned before which involved the heterogeneous constituents rendering the EVs either immunostimulatory or immunosuppressive effect, may lead to side effects such as immune, inflammatory, or even toxic responses. As such, more efforts need to be made to clarify the total compositions, evaluate the immunogenicity, and ensure the safety of EVs inside the human body before reaching clinical use.

### Isolation, purification and large-scale production

To eventually achieve the commercialization and clinical translation of EVs, several critical issues on isolation, purification, and large-scale production of the currently available preparation techniques need to be solved. For instance, the most commonly employed approaches such as differential ultracentrifugation and sequential ultrafiltration usually exhibit crucial limitations of time-consuming procedures, compromised purity, poor reproducibility, and low production yield, which would greatly hinder the massive and stable manufacture of EVs for clinical trials. Despite the recently developed commercialized isolation kits for the harvest of EVs, the low efficiency and high expense of such kits may cause an enormous obstacle for EV-based nanotherapeutics for further widely practical application.

Therefore, much diligence should be taken in developing a reproducible standardized isolation technique that can mass-produce high-purity EVs at low cost, making EVs become authentic clinically settled therapeutics.

Overall, the utilization of EVs as bioactive nanotherapeutics for regenerative medicine is still in the stage of preliminary development. Especially when compared to stem cell therapy, EVs-based therapy has more challenges to be addressed, including 1) EVs cannot respond to the pathological environmental stimuli as stem cells do, leading to a lack of plasticity for EVs in the treatment of various diseases; 2) current immature large-scale production processes, additional isolation procedures and optimal quality control of EVs make them much more expensive than stem cell therapies, greatly hindering the clinical translation of EVs; 3) the clinical studies of stem cells cannot be simply transferred to the stem cell-secreted EVs because the therapeutic mechanisms of EVs in some specific diseases may be completely different from their source stem cells. Although challengeable in principle, with continued advances in expanding knowledge of EV biology and therapeutic mechanism as well as with strong cooperation in multidisciplinary fields of bioengineering, chemistry, material science, nanotechnology, clinical medicine, and industry, we believe that not only more potential in tissue engineering and regenerative medicine of EVs will be discovered, but also it may ignite further explorations and inspiration for clinical applications of safe and efficient nanotherapeutics in various biomedical fields in the near future.

## Figures and Tables

**Table 1:** Current major isolation technology of EVs

Isolation technique	Principle	Advantage	Disadvantage	Refs
Differential ultracentrifugation	To stepwise remove different extracellular components through specific centrifugal forces	The gold standard isolation approach; suitable for large-volume specimens, especially cell culture supernatant, urine, *etc.*; low cost	Time and labor consuming; expensive equipment requirements; risk of contamination by protein and impurities; low recovery rate; potential mechanical damage	49-52
Density-gradient centrifugation	To stratify extracellular components in the position of the medium with similar density by gravitational or centrifugal force fields	High purity of EVs; preservation of EV activity	Time and labor consuming; expensive equipment requirements; large amount of pre-work and complicated steps; low EV yield	50,52,56
Ultrafiltration	To selectively separate EV samples using different molecular weight cut-offs ultrafiltration membranes	Short operation time; low equipment cost; suitable for large-volume specimens	Labor consuming; moderate purity; possible loss due to clogging membrane; potential physical changes induced by shear stress; low EV yield	52,57
Immunoaffinity capture	Based on specific binding between biomarkers such as surface antibodies of EVs and antibody-recognized ligands immobilized on beads or filters	High specificity and purity; easy operation; no potential mechanical damage	High-cost; low efficiency; environment-susceptive ligands activity susceptible to processing environment; low EV processing volume and yields	50,52-54
Size-exclusion chromatography	Based on the different sizes of EVs which exhibit various elution times passing through porous resin particles	High purity; easy operation; good reproducibility; preservation of the native state of EVs; suitable for most of downstream analysis	Time consuming; relatively high preparation cost	49,50,52,58
Polymer Precipitation	Using highly hydrophilic water-excluding polymers to reduce the solubility of EVs and then precipitating EVs by low-speed centrifugation	Short operation time; preservation of the native state of EVs; high yield; easy accessibility	Contaminations of non-EVs; affection on downstream analysis and quantification of EV samples	50,52,53
Microfluidic techniques	Based on parameter differences of the microfluidic channels, physicochemical or biological variations of the EVs, and even additional field forces	Low sample consumption; fast processing time; high sensitivity; suitable for quantitative detection of scarce samples	Low sample capacity; nonspecific binding	59-62

**Table 2:** Regenerative mechanisms of specific components in EVs

Regenerative cargo	Nanovesicles	EV source cell types	Resulting therapy effects	Intracellular signaling pathways	Disease model	Refs
miR-125b	Exosomes	Chorionic plate-MSCs	Stimulation of proliferation, inhibition of fibrosis	Hedgehog signaling↓	Liver fibrosis	70
miR-210	EVs	MSCs	Increased the proliferation, migration and tube formation capacity of HUVECs	Efna3↓	Myocardial infarction	69
miR-21	Exosomes	Human endometrium-MSCs	Antiapoptotic and angiogenic	Phosphatase and tensin homolog ↓and Akt pathway↑	Myocardial Infarction	103
miR-23a, miR-145	Exosomes	Umbilical cord-MSCs	Reduced scar formation and myofibroblast accumulation	TGF-β2, TGF-βR2, and Smad2↓	Skin-defect model	68
miR-146a	Microvesicles	Bone marrow-MSC(BM-MSCs)	Promote allogeneic kidney graft survival	IL-12 mRNA↓	Kidney transplant model	104
miR-31	Microvesicles	Adipose-stem cells	Promote angiogenesis	Hypoxia inducible factor -1↓	Ischemic cardio- and cerebrovascular diseases	105
miR-133b	Exosomes	Multipotent-MSCs	Promotes neural plasticity and functional recovery	Connective tissue growth factor and *ras* homolog gene family member A↓	Stroke	106
miR-328-3p	Apoptotic bodies	BM-MSCs	Maintain mesenchymal stem cell homeostasis and ameliorate osteopenia	Axin 1↓ and wnt/β- catenin↑	Osteoporosis	107
miR-23a-3p	sEVs	Human umbilical cord-MSCs (HUC-MSCs)	Proliferation, migration, differentiation of chondrocytes	Pten↓ Akt↑	Cartilage defects	108
IGF-1R mRNA	Exosomes	BM-MSCs	Proliferation of proximal tubular epithelial cell		Acute kidney injury	109
HGF-mRNA	Microvesicles	HUC-MSCs	Acceleration of tubular cell dedifferentiation and growth, enhance HGF expression	Erk1/2↑	Acute kidney injury	66
KGF- mRNA	Microvesicles	BM-MSCs	Promote angiogenesis, inhibiting apoptosis, anti- inflammation		Acute lung injury	74
VEGF, IGF-1, FGF- mRNA	Microparticles	Kidney-MSC	Improving proliferation		Acute kidney injury	110
IL-10 mRNA	EVs	BM-MSC, HUC-MSC	Anti-inflammation	IL-10/IL-10R1R2↑	Acute cisplatin injury	111
PDGF-BB	Apoptotic bodies	Osteoclast	Induced endothelial progenitor cell differentiation	Receptor activator of nuclear factor κB (NFκB) ligand reverse signaling↑	Bone defect model	112
Glutathione peroxidase1	Exosomes	Human umbilical cord MSC	Reduced oxidative stress and apoptosis	Erk1/2 and Bcl-2↑, IKKβ/NFκB/casp-9/-3↓	Liver failure	113
Specificity protein1	EVs	Human-induced pluripotent stem cell-MSCs	Transcriptional activating of sphingosine kinase 1 and inhibiting necroptosis	Sphingosine kinase1↑	Renal ischemia-reperfusion	114
HSP70	Microvesicles	Human embryonic neural stem cell	Anti-apoptosis of HL-1 cardiomyocytes	pAkt/mTOR↑	Myocardial reperfusion injury	65
STAT3	Exosomes	Adipose-stem cells	Improved metabolic homeostasis, and resistance to obesity progression	Arginase 1↑	Obesity	67
14-3-3 ζ protein	Exosomes	keratinocyte-like cells	Anti-fibrogenic	Matrix metalloproteinase 1↑	Healing wounds	115
Neprilysin	Exosomes	Adipose-MSCs (AD-MSCs)	Decrease both secreted and intracellular β-amyloid peptide levels in the N2a cells	Amyloid β↓	Alzheimer's disease	116
Neuregulin 1	EVs	Adipose Stem Cells	Proliferation and differentiation, anti-apoptotic	Erk1/2 and Bcl-2↑, ErbB system↑	Hind limb ischemia	117

**Table 3:** Engineering strategies of the modified EVs for biomedicine

Strategies	Method	Principle	Advantages	Disadvantages	Main applicable molecular types	Refs
Endogenous cargo loading strategy	Genetic engineering strategy	Genetically manipulate the biosynthesis process of donor cells	Suitable for all types of EVs and applicable to all types of RNA	Nonspecific loading mechanism; low drug loading efficiency	RNA and proteins, such as miR-133b, miR-122, FOXF, GATA-4	122,144,145
	Chemical engineering strategy	Apply covalent chemical reactions, commonly combine metabolic engineering and click chemistry	Suitable for various molecules; new surface compositions can be added	Destroy the integrity and function of the membrane	Various chemical molecules, such as PEGylated hyaluronic acid, Ac4ManNAz, AHA	125,146,147
	Physical engineering strategy	Direct physical force to the donor cells or regulation of cell culture conditions	Simple; easy to operate	Low drug loading efficiency; potential cytotoxicity to the donor cells, nonspecific loading mechanism	Nucleic acid, protein, growth factors and small molecules, such as HIF-1α, VEGF, miR-146a, catalase	127,135,148,149
Exogenous cargo loading strategy	Co-incubation	Direct co-incubation of EVs with various compounds at different conditions	Simple; inexpensive	More suitable for hydrophobic molecules	Chemotherapy drugs, such as curcumin, DEX	150
	Electroporation	Use high-intensity short-duration voltage to generate transient permeable pores on the surfaces of EV membranes	Suitable for all types of EVs; applicable to biomacromolecules with large size	Affect the zeta potential and colloidal stability of EVs; EVs aggregation trend	Small molecule drugs or biomacromolecules, such as miR-21a, ADK siRNA, antisense oligonucleotides	130-132,151,152
	Sonication	Use mechanical shear force to break the membrane integrity of the EVs	High drug loading efficiency	Compromised membrane integrity; cargos may adhere to the EV outer layer	Small molecule drugs or enzymes, such as TPP1, paclitaxel	153,154
	Mechanical extrusion	Use syringe-based mini-extruder to extrude the mixture of the cargos and EVs	Simple; high drug loading efficiency	Compromised membrane integrity	Different synthetic nanoparticles such as IONPs, AuNPs	134,155
	Freeze-thaw cycles	Use freeze-thaw cycles to alternately form ice crystals and water molecules within lipid bilayer membranes, resulting in disrupting EV membranes	No external mechanical force damage; no chemical contamination of the EV membranes	Complicated operation; EVs aggregation trend	Chemotherapeutic drugs and biomacromolecules, such as curcumin, neprilysin, catalase	135,137,156
	Direct EV membrane modification	Covalent and noncovalent modification of EVs membrane	Efficiently endow the modified EV with additional functions	Long-term biocompatibility, stability, and safety need to be further clarified	Small molecule drugs or biomacromolecules, such as aptamer, peptide	27,141,157

## Abbreviations

EVs: extracellular vesicles
MVs: microvesicles
miRNA: microRNA
mRNA: messenger RNA
ESEs: early-sorting endosomes
LSEs: late-sorting endosomes
ILVs: intraluminal vesicles
BM-MSCs: human bone marrow mesenchymal stem cells
KGF: keratinocyte growth factor
TGF: transforming growth factor
TCR: T cell receptor
TLR4: Toll-like receptor 4
SCI: spinal cord injury
BDNF: brain-derived neurotrophic factor
FGF-1: fibroblast growth factor-1
GDNF: glial cell-derived neurotrophic factor
IGF-1: insulin-like growth factor-1
NGF: nerve growth factor
HGF: hepatocyte growth factor
SDF1: stromal-derived growth factor-1
ROS: reactive oxygen species
Nrf2: nuclear-related factor 2
BM-MSCs: Bone marrow-MSC
HUC-MSCs: Human umbilical cord-MSCs
NFκB: nuclear factor κB
AD-MSCs: Adipose-MSCs
MGE: metabolic glycoengineering
DBCO: dibenzocyclooctyne
NPs: nanoparticles
TBI: traumatic brain injury
ESC-sEVs: embryonic stem cell derived small EVs
MNV: magnetic nanovesicle
IONP: iron oxide nanoparticles
CVD: cardiovascular diseases
GMNP: grafted magnetic nanoparticle
hMSCs: human mesenchymal stem cells
Ti-scaffolds: titanium alloy scaffolds
EXO-D10 and EXO-D15: exosomes pre-differentiated for 10 and 15 days
Ems: exosome mimetics
CKD: chronic kidney disease
IRI: ischemia/reperfusion injury
CCT2: Chaperonin Containing TCP1 Subunit 2
PDRN: polydeoxyribonucleotide
TNF-α: tumor necrosis factor-α
IFN-γ: interferon-γ
IL4: interleukin 4
CXCL12: C-X-C motif chemokine ligand 12
COVID-19: Corona Virus Disease 2019
